# Managing terminal restlessness, anxiety, and distress during the dying process with Yintang (EX-HN 3) point acupuncture or acupressure: a case series of 19 palliative care patients from a hospital in Germany

**DOI:** 10.11604/pamj.2022.42.99.32513

**Published:** 2022-06-07

**Authors:** Angeliki Konstantinou, Lykourgos Christos Alexakis

**Affiliations:** 1 Palliative Medicine Unit, Department of Oncology and Hematology, Nordwest Hospital, Frankfurt, Germany,; 2 Independent Researcher, Andersenweg 10, 60431, Frankfurt, Germany

**Keywords:** Acupuncture, acupuncture therapy, acupressure, palliative care, palliative medicine, terminal care, Yintang point, Yintang (EX-HN 3)

## Abstract

This is a case series report of 19 palliative care patients where 23 acupuncture sessions were performed which included Yintang (EX-HN 3) acupuncture or acupressure for the relief of terminal restlessness, anxiety or psychological distress present during the dying process. There was an observable relief from the restlessness, anxiety and distress both in sessions where only Yintang (EX-HN 3) point acupuncture or acupressure was performed (observed in 10 out of 11 sessions) as well as in sessions where Yintang (EX-HN 3) point acupuncture or acupressure was performed together with additional interventions, such as other points acupuncture, ear acupuncture or benzodiazepine treatment (observed in 10 out of 12 sessions). In total relief was observed in 20 out of 23 sessions (86.9%). A hypothesis that might worth further testing is whether Yintang (EX-HN 3) acupuncture or acupressure has an anxiolytic, tranquillising or sedative effect in dying patients. If confirmed this could be potentially useful in the fields of palliative care or disaster/triage medicine.

## Introduction

Complementary and alternative medicine has been used in palliative medicine patients especially in cases where symptoms are inadequately controlled with conventional therapies. In many studies a short-term improvement of symptoms was shown [1]. Acupuncture is among the most researched complementary medicine therapies and its evidence base is quickly growing. As it is safe, acupuncture is increasingly recommended by many experts for the treatment of symptoms in oncology and palliative medicine [2]. Reaching a calm state can be achieved with the use of complementary medicine techniques including acupuncture [3]. On the other hand, the effect of acupuncture on psychological well-being of cancer patients has been reviewed only in two systematic reviews and no significant positive effect was identified [4]. There is a need for more research on the effects of acupuncture on the psychological state of palliative care patients.

One of the authors (AK) during her work in a major palliative care unit provided acupuncture treatment, for the management of anxiety, distress and other specific symptoms of terminally ill patients, complementary to standard palliative care. Quickly a pattern emerged where patients showed considerable relief from the anxiety and distress present during the dying process - often followed by falling asleep - after acupuncture or acupressure in Yintang (EX-HN 3) point. Additionally in many cases death occurred peacefully shortly thereafter, e.g within a few hours. A decision was made to collect and review all these cases. The aim was to document any findings which might help generate a new hypothesis for further testing. The objective of this case series was to identify the proportion of acupuncture or acupressure sessions during which improvement of terminal restlessness, anxiety and distress was observed, among the palliative medicine patients who were treated with acupuncture or acupressure in Yintang (EX-HN 3) point.

## Methods

This is a case series report, a descriptive study based on the retrospective collection of anonymised data from patient files. From the patients admitted in the palliative medicine unit of the Nordwest hospital in Frankfurt am Main, Germany from July 2019 till June 2021, all 19 patients who received Yintang (EX-HN 3) point medical acupuncture or acupressure complementary to their standard medical treatment were included. In some of these patients, additional acupuncture points were used depending on their specific symptoms. Common medical acupuncture needles were used (B type with 0.20mm diameter and 15mm length) and each acupuncture session lasted approximately 20-30 minutes. In patients with bleeding diathesis or with an increased risk of bleeding, as judged by the doctor, acupressure was performed instead with a duration of approximately three minutes. All sessions were performed by the same medical doctor, an anaesthesiologist trained in medical acupuncture, who is one of the authors (AK). In all 19 patients Yintang (EX-HN 3) acupuncture or acupressure was performed for the treatment of terminal restlessness, anxiety or psychological distress present during the dying process. The outcomes were collected from the patient files, which in some cases included also patient feedback following the intervention, and direct observations captured by the doctor performing the session. In this case series improvement was defined as an observation of either the patient falling asleep during or shortly after the session, or of relief of patient´s anxiety and distress or of a temporary decrease of seizures (for the 3 cases where seizures were present). The acupuncture points that were used in the patients included in this case series are described in detail in Table 1 [5-8].

**Table 1:** acupuncture points used for the management of terminal restlessness, anxiety and distress during the dying process in a sample of 19 palliative care patients from Nordwest hospital in Frankfurt, Germany

Acupuncture point/ (abbreviation)	Location	Indications
Yintang (EX-HN 3)	On the anterior midline between the eyebrows.	Psychological/ mental restlessness
Pericardium 6 (PC 6)	Anterior aspect of forearm, three fingers proximally from the base of the palm between the two tendons.	Nausea, vomiting
Spleen 9 (SP 9)	In the depression at the interior border of the medial epicondyle of the tibia.	Oedema
Urinary bladder 40 (BL 40)	Midpoint of the transverse crease of the popliteal fossa.	Back pain
Large intestine 4 (LI 4)	Between the 1^st^ and 2^nd^ metacarpal bones (location depressed as a valley).	Analgesia
Governor vessel 20 (GV 20)	At the vertex.	Anxiety
Conception vessel 17 (CV 17)	Middle part of the chest.	Dyspnoea
Ear: Shen men (55)	At the cranial junction of the triangular fossa with the superior crus of antihelix, between the 1^st^ and 2^nd^ third, starting from the tip of the triangular fossa.	Tranquillising effect, analgesia
Ear: stomach (87)	At the floor of concha around the root of helix.	Gastritis, nausea
Ear: lung (101)	Central in the inferior hemiconcha.	Lung disease
Ear: vegetative 1 (51)	On the inferior crus of the antihelix under the protruding helix rim.	Spasmolytic, relaxation

## Results

Our sample of palliative care patients included 14 patients with advanced stage cancer, 3 patients with haematological malignancies (one chronic myelomonocytic leucemia/CML with myelodysplastic syndrome/MDS, and two cases of acute myeloid leucemia /AML), 1 patient with subarachnoid haemorrhage (SAH) and 1 patient with chronic kidney disease and hepatic cirrhosis. These 19 patients had received in total 23 acupuncture or acupressure sessions in which Yintang (EX-HN 3) point acupuncture or acupressure was included (Table 2). From the 19 patients, 4 received only Yintang (EX-HN 3) point acupuncture, 4 received only Yintang (EX-HN 3) point acupressure, while 11 received Yintang (EX-HN 3) point acupuncture or acupressure together with additional interventions such as acupuncture in other acupuncture points, ear acupuncture or benzodiazepine treatment (Table 3).

**Table 2:** details of patients cases from Nordwest hospital in Frankfurt, Germany from July 2019 to June 2021 (N=19)

N	Age	Sex	Diagnosis	Symptoms	Intervention	Patient outcome
1	61	M	Non-small cell lung adenocarcinoma, retroperitoneal metastases, pneumothorax, empyema.	Dyspnoea, anxiety, fear.	EX-HN3 acupressure, 55, 101	Fell asleep.
				Dyspnea, fear (2^nd^ day).	EX-HN3	Relief. Death in minutes.
2	73	M	Prostate cancer with bone metastases. P. Jirovecii pneumonia, aspiration pneumonia, pleural effusions under drainage. On antibiotics including cotrimoxazole.	Anger, nausea, vomiting.	EX-HN3, PC6, 55, 87	Fell asleep.
				Patient request (2^nd^ day).	EX-HN3, PC6, 55, 87	Unknown
				Dyspnoea (3^rd^ day).	EX-HN3	Slept. Died in 30 min.
3	71	M	CML, MDS, cirrhosis, renal failure, ascites.	Dyspnoea, hypotension.	EXHN3, SP9, BL40, 101	Slept. Died in 5 min.
4	69	F	Gastric adenocarcinoma, peritoneal metastases, ileus.	Severe anxiety, fear of death (on midazolam)	EX-HN3 acupressure	Fell asleep. Died 6 hours later.
5	85	F	Acute myeloid leukeumia (AML) (M1), lung emphysema, pneumonia.	Dyspnoea, O_2_ sat 88%, fear of death.	EX-HN3	Slept. Died in 20 hours.
6	66	F	Non-small cell lung adenocarcinoma, metastases (brain, bone).	Dyspnea, anxiety and distress despite sedation.	EX-HN3 acupressure	Became calm, slept. Died in 1 hour.
7	67	F	Metastatic colon carcinoma.	Dyspnea, distress, fear.	EX-HN3 acupressure	Calmed. Died in 2 hours.
8	71	F	Metastastic serous ovarian carcinoma.	Anxiety, diffuse fear.	EX-HN3	Slept. Died in 2 hours.
9	70	F	SAH, brain infarcts, pneumonia.	Restlessness on sedation.	EX-HN3	Died in a few minutes.
10	75	F	Gastric ulcer bleeding, cirrhosis, atrial fibrilaton (apixaban), stage 3 CKD.	Seizures on midazolam, (last dialysis 7 days ago).	Clonazepam/ diazepam perf. EX-HN3 acupressure.	Seizures decreased for few min. Died in 24 hrs.
11	83	F	Adenocarcinoma of oesophagogastric junction causing subtotal stenosis, hepatic/peritoneal metastases, diabetes mellitus, chronic kidney disease.	Nausea, severe anxiety, alert and wanted to die.	EX-HN3, PC6 midazolam perfusion	Fell asleep.
				Anxiety (on midazolam).	EX-HN3	Calmer. Died in 24 hrs.
12	85	F	AML, MDS, fungal pneumonia, fall with cerebral contusion and infarct.	Pain and distress (painful expressions, moaning).	EX-HN3 acupressure	Patient relaxed. Died 3 hours later.
13	58	F	Ovarian carcinoma, liver metastases, brain infarcts, global aphasia.	Dyspnea, anxiety (on fentanyl).	EX-HN3, 55	Died two minutes later
14	59	M	Pancreatic adenocarcinoma, peritoneal metastases, ascites.	Dyspnea, restlessness.	EX-HN3, 55, 51	Became calmer. Died in one hour.
15	48	F	Cervix carcinoma with hepatic metastases.	Epileptic seizures (dying patient).	Midazolam 4 mg EX-HN3 acupr, LI4	Seizures stopped. Died 9 hours later.
16	37	F	Pancreatobiliary adenocarcinoma, liver metastases, stomach outlet stenosis.	Seizures unresponsive to benzodiazepines.	EX-HN3, LI4, 55	Seizures stopped for a while. Died in one hour.
17	60	F	Non-small cell lung cancer, metastases (pleural, mediastinal), effusion.	Severe dyspnea and breathlessness, nausea.	EX-HN3, GV20, CV17	Patient relaxed. Died in 24 hours.
18	86	F	Non-small cell lung adenocarcinoma, aspiration pneumonia.	Dyspnea and coughing.	EX-HN3, GV20, CV17	Dyspnea decreased. Died in two days.
19	87	F	Gastric carcinoma, bone metastases.	Restlessness, dyspnea.	EX-HN3	Calmed. Died in 1 hour.

**Table 3:** details of sessions where Yintang (EX-HN 3) acupuncture or acupressure was performed together with additional interventions

Interventions performed	Number of sessions	Number of sessions with observed improvement	Case number
Yintang acupressure, ear acupuncture: Shen men, lung	1	1	Patient 1
Acupuncture: Yintang, PC 6, ear acupuncture: Shen men, stomach	2	1	Patient 2
Acupuncture: Yintang, SP 9, BL 40, ear acupuncture: lung	1	1	Patient 3
Yintang acupressure, diazepam and clonazepam perfusion	1	1	Patient 10
Acupuncture: Yintang, PC 6, midazolam perfusion	1	1	Patient 11
Acupuncture: Yintang, ear acupuncture: Shen men	1	0	Patient 13
Acupuncture: Yintang, ear acupuncture: Shen men, vegetative 1	1	1	Patient 14
Acupuncture: Yintang, LI 4, midazolam 4 mg intravenously	1	1	Patient 15
Acupuncture: Yintang, LI 4, ear acupuncture: Shen men	1	1	Patient 16
Acupuncture: Yintang, GV 20, CV 17	2	2	Patients 17, 18

In total in 20 out of the 23 acupuncture or acupressure sessions (86.9%) in which Yintang (EX-HN 3) point was included there was an observed improvement of the patient´s terminal restlessness, anxiety or psychological distress, meaning that the patient fell asleep or the anxiety and distress was relieved or the seizures temporarily decreased. For 3 out of the 23 sessions the data captured in file were not enough to confirm an observed improvement (patients 13, 9 and the second session of patient 2). Improvement was observed in 6 out of the 7 sessions (85.7%) where only Yintang (EX-HN 3) point acupuncture was performed and in 4 out of 4 sessions (100%) where only Yintang (EX-HN 3) point acupressure was performed. Concerning the sessions where Yintang (EX-HN 3) point acupuncture or acupressure was performed together with additional interventions (such as acupuncture in additional acupuncture points, ear acupuncture, or benzodiazepine treatment) there was an observed improvement in 10 out of 12 sessions (83.3%). If we consider exclusively the sessions where only acupuncture or acupressure at Yintang (EX-HN 3) point was done, there was an observable relief from the terminal restlessness, anxiety and distress during the dying process in 10 out of the 11 sessions (90.9%) performed (Table 4).

**Table 4:** summary of outcome results following acupuncture or acupressure in Yintang (EX-HN 3) point for the management of terminal restlessness, anxiety and distress during the dying process in a sample (case series) of 19 palliative care patients from Nordwest hospital in Frankfurt, Germany, from July 2019 to June 2021

	Number of patients	Number of sessions	Number of sessions with observed improvement*	Percentage of sessions with observed improvement
**Yintang (EX-HN 3) acupuncture only**	4	7	6	85.7%
**Yintang (EX-HN 3) acupressure only**	4	4	4	100%
Yintang (EX-HN 3) acupuncture or acupressure with additional interventions**	11	12	10	83.3%
**Total**	19	23	20	86.9%

* Patient fell asleep or anxiety/distress was relieved or seizures temporary decreased. The sessions in which such an improvement could not be confirmed by the available data were not included. **Acupuncture in additional points, ear acupuncture or benzodiazepine treatment.

## Discussion

Acupuncture has been used in cancer and palliative care patients for the management of symptoms related to the disease itself (pain, fatigue, dyspnoea, anxiety) or its treatment (nausea, vomiting) and can improve the severity of breathlessness in patients with cancer or COPD [9,10]. Anxiety is a condition often present in terminally ill patients and periods of fear and distress are observed as death approaches [11,12]. Yintang (EX-HN 3) point acupuncture or acupressure was shown before to decrease the anxiety levels preoperatively in patients waiting for neurosurgery or gastrointestinal tract surgery [13,14]. There is also a case report of successful management of restlessness and agitation in a three months old paediatric patient with acupuncture sessions which included - among others - Yintang (EX-HN 3) and Shen Men (ear) points. Subsequently sedative medications were discontinued [15]. Nevertheless to our knowledge this is the first report on the use of Yintang (EX-HN 3) point acupuncture or acupressure for the management of terminal restlessness, anxiety and distress in dying palliative care patients. In our sample of patients in 12 out of 23 sessions many different interventions were used together with acupuncture or acupressure in Yintang (EX-HN) point which makes evaluation of their outcomes more difficult. On the other hand, in 11 sessions only acupuncture or acupressure at Yintang (EX-HN 3) point was done and in 10 of them (90.9%) there was an observable relief from the anxiety and distress present during the dying process. As a result, the hypothesis whether Yintang (EX-HN 3) acupuncture or acupressure has an anxiolytic or sedative effect in dying patients might worth further testing. The location of Yintang (EX-HN 3) can be easily identified and can be used with minimal equipment and training, also by medical and paramedical staff not specialized in acupuncture. Such an intervention could be a cost effective one to further explore and consider in the field of palliative medicine, as well as in situations where access to palliative care services is restricted or non-existent (e.g. limited resources settings, remote geographical locations, disaster related triage situations).

**Limitations:** being a case series, there are many limitations in this study and no causal inferences can be made. There is no control group to compare the outcomes and the study is based on retrospective collection of data which can lead to bias due to insufficient records. Furthermore, only a small sample of patients is included in this series so it is prone to selection and measurement bias. On the other hand, a case series might have high external validity and relevance as the patients included are more diverse and more representative of the routine clinical practice [16].

## Conclusion

In our series of palliative care patients, relief from terminal restlessness, anxiety and distress present during the dying process was observed in 20 out of 23 sessions (86.9%) where acupuncture or acupressure at Yintang (EX-HN 3) point was performed alone or with additional interventions. A hypothesis that might worth testing in further studies is whether Yintang (EX-HN 3) point acupuncture or acupressure has an anxiolytic, tranquillizing or sedative effect in dying patients. If confirmed this could be potentially useful in the fields of palliative care, disaster and triage medicine.

### What is known about this topic

- Acupuncture has been used for symptomatic management in palliative care;
- Anxiety and fear is often present in terminally ill patients;
- Yintang (EX-HN 3) point acupuncture or acupressure decreased the anxiety levels in patients before an operation.

### What this study adds

- Relief from terminal restlessness, anxiety and distress present during the dying process was observed in 20 out of 23 sessions (86.9%) where Yintang (EX-HN 3) point acupuncture or acupressure was performed alone or with additional interventions, in a series of palliative care patients;
- A hypothesis that might worth further testing is whether Yintang (EX-HN 3) point acupuncture or acupressure has an anxiolytic, tranquillising or sedative effect in dying patients.
